# Anal endosonographic assessment of the accuracy of clinical diagnosis of obstetric anal sphincter injury

**DOI:** 10.1007/s00192-021-05044-x

**Published:** 2021-12-31

**Authors:** Angharad Jones, Linda Ferrari, Paula Igualada Martinez, Eugene Oteng-Ntim, Alison Hainsworth, Alexis Schizas

**Affiliations:** 1 Guy’s and St Thomas NHS foundation Trust, Westminster Bridge Road, London, SE1 7EH UK; 2 King’s College London, Great Maze Pond, London, SE1 1UL UK

**Keywords:** Anal incontinence, Endoanal ultrasound, Obstetric anal sphincter injuries, Pelvic floor

## Abstract

**Introduction and hypothesis:**

Obstetric anal sphincter injuries (OASIS) are a common cause of maternal morbidity with an overall incidence in the UK of 2.9% (range 0–8%). They can cause a range of physical symptoms and psychological distress. This study aims to assess the accuracy of clinical diagnosis of OASIS using endoanal ultrasound (EAUS) and the correlation between confirmed injury and change to anorectal physiology squeeze pressure and the incidence of bowel symptoms.

**Methods and materials:**

Retrospective study of prospectively collected data from 1135 women who attended the Third- and Fourth-Degree Tears Clinic at our institution, 12 weeks post-delivery, between June 2008 and October 2019.

**Results:**

OASIS was confirmed in 876 (78.8%) women and 236 (21.3%) had no injury. Of the women who underwent anorectal physiology, 45.6% had a mean maximal resting pressure below the normal range and 68.8% had a mean incremental squeeze pressure below normal. Women with confirmed OASIS had significantly lower pressures (*p* < 0.001) than those without a confirmed sphincter injury. Three hundred ninety-three (34.8%) women reported bowel symptoms, with those with endosonographic evidence of injury more likely to develop flatus incontinence.

**Conclusion:**

Of the women in this study with a suspected OASIS, 21.2% could be reassured that they did not have an injury. This information is useful for women considering future mode of delivery. Those with confirmed injury are more likely to complain of flatus incontinence and have reduced anal sphincter pressures.

## Introduction

### Background

Obstetric anal sphincter injuries (OASIS) are a significant cause of maternal morbidity, which can lead to serious physical, social and psychological consequences. A 2014 study across 215 maternity units found the overall incidence in the UK to be 2.9% (range 0–8%) with 6.1% of these occurring in primiparous women and 1.7% in multiparous women [1]. The incidence rate of OASIS in England was found to have increased from 1.8% to 5.9% between 2002 and 2012. While there was an increase in certain risk factors found over this time frame, this is unlikely to account for the magnitude of the increase of OASIS. It is suggested that improvement in the recognition and reporting of tears, following the implementation of a standardized guidelines and classification of perineal tears by RCOG in 2001, is the most likely explanation for this [2]. Perineal obstetric injuries can be grouped into first-, second-, third- and fourth-degree tears based on Sultan’s classification, with third- and fourth-degree tears considered obstetric anal sphincter injuries [3].

### Adverse effects

Sixty to 80% of women with a clinical diagnosis of OASIS are asymptomatic at 12 months [4]; however, some of them will develop pelvic floor symptoms that only become apparent years post obstetric injury [5]. The most distressing adverse effect of OASIS is anal incontinence (AI), which may be short and/or long term and vary in severity [6]. AI is defined as involuntary leakage of flatus, liquid and/or solid stool [7]. There is a significant association between OASIS and anal incontinence [7, 8]. Women who sustain OASIS are 2.66 times more likely to have anal incontinence than women who do not have OASIS (pooled OR, 2.66; 95% CI, 1.77–3.98) [8]. OASIS also significantly affects women’s psychological well-being and quality of life. It has been suggested that women with AI and OASIS have a specific syndrome, the ‘OASIS syndrome’ [9].

There is an increased risk of anal incontinence in women with fourth-degree tears compared with those with third-degree tears but conflicting evidence when comparing 3a, 3b and 3c tears [10, 11]. Other pelvic floor-associated symptoms include urinary incontinence, sexual dysfunction, perineal pain, pelvic organ prolapse and rectovaginal fistulas [6, 12].

### Clinical diagnosis/assessment

Following all vaginal deliveries, women are examined for perineal trauma; this includes a rectal examination. It can, however, be difficult to make an accurate assessment at the time of delivery. Therefore, endoanal ultrasound (EAUS) is accepted as the gold standard tool to assess the integrity of the anal sphincter complex following a suspected OASIS [13, 14]. Besides identifying injury to the anal sphincters, EAUS can assess the ancillary pelvic floor muscles (transverse pereni, puborectalis and puboanalis) and the length of the perineal body. This technique was first described by Law and Bartrum in 1989 and is simple, quick and well tolerated. A 2D or 3D probe is inserted into the anus and used to provide a 360-degree anatomical image of the anal sphincter complex. It has been equally accurate as MRI (magnetic resonance imaging) but is both cheaper and more accessible and is therefore the preferred method of diagnosis [15].

Anorectal physiology (ARP) encompasses several tests, including anal manometry, which is used to objectively assess the function of the anal sphincter complex. A pressure-sensitive catheter is inserted into the anal canal, and the patient is asked to carry out certain manoeuvres to assess the maximal resting and incremental squeeze pressures [16].

St Mark’s Incontinence Score (SMIS) is a validated questionnaire used to screen for and assess the severity of faecal incontinence and might be used following obstetric anal sphincter injury. Possible scores range from 0 to 24. It has been shown to correlate well with patients' own perception of their symptoms and quality of life [17, 18].

This study assesses the accuracy of clinical diagnosis of obstetric anal sphincter injuries using EAUS, the correlation between confirmed injury and change in anorectal physiology squeeze pressure and the incidence of bowel symptoms. Although several studies have recently examined the accuracy of clinical diagnosis of OASIS, these have used alternative methods—transperineal and translabial—rather than the gold standard investigation EAUS used in our study [19, 20]. To the best of our knowledge, this is the first paper to examine a sample size of this magnitude. Of the studies that have examined the accuracy of clinical diagnosis of OASIS, most have approached it by reviewing how many obstetric tears are missed clinically, then later diagnosed on ultrasound. In contrast, our study looks at women with clinically suspected tears who then have this confirmed or refuted on EAUS. This allows those women who do not have an injury confirmed on EAUS to make a shared informed decision about future modes of delivery and not exclude vaginal deliveries unnecessarily.

## Materials and methods

This is a retrospective review of prospectively collected data to assess the accuracy of clinical diagnosis of OASIS using EAUS as well as the correlation between confirmed injury and anorectal physiology results and symptoms.

In our institution, all women who are suspected of sustaining an OASIS following vaginal delivery are referred to a dedicated Third and Fourth Degree Tears Clinic 3 months post-partum. Between June 2008 and October 2019, 1135 women were assessed in our clinic.

The clinic runs weekly and is staffed by a multidisciplinary team, including a consultant obstetrician, a physiotherapist, a continence nurse specialist and a clinical scientist. It is also overseen by a colorectal consultant with an interest in pelvic floor. During attendance at clinic, a full history was taken, and patients were asked about bowel, urinary and sexual symptoms. Patients were asked to complete a validated and standardized questionnaire (St Marks Faecal Incontinence Score) to assess for anal incontinence. Besides, three-dimensional EAUS and ARP studies were performed.

Anal sphincter injuries clinically diagnosed at the time of delivery were repaired by obstetric registrars or consultants, who were appropriately trained in OASIS repair, according to the hospital protocol. The hospital has a standardized protocol for primary repair, management and follow-up of women who have sustained OASIS, which is in line with the Royal College of Obstetricians and Gynaecologists guidance [4].

Of the 1135 women who attended clinic, 1112 underwent EAUS. This was performed in the prone position using a B&K Medical 2050 three-dimensional EAUS probe. ARP was performed on 1018 of these women using Medical Measurement Systems water perfused system and an 8-channel radially arranged catheter to assess mean maximal resting pressure and mean incremental squeeze pressure.

The data were analysed using Microsoft Excel and SPSS 27 (Statistical Package for Social Sciences). Univariate analysis was used to obtain frequencies, means and standard deviations to describe demographic data. Independent t-tests were used to analyse parametric data and the Mann Whitney U test to analyse non-parametric data. A multinomial logistic regression analysis was performed to evaluate the differences in risk of developing bowel symptoms. A *p* value < 0.05 was considered statistically significant.

## Results

### Anal endosonography

One thousand one hundred twelve women who were clinically diagnosed with OASIS underwent EAUS. Results confirmed anal sphincter injury in 876 (78.8%) women, and 236 (21.2%) of women were found to have no anal sphincter injury.

Of the 876 women with a confirmed OASIS, 347 had persistent defects to the external anal sphincter; of these, 228 (26.0% of 876) had a persistent defect to the external anal sphincter only, classified as a grade 3a or 3b tear.

Two hundred forty (27.4%) women had a persistent defect to the internal anal sphincter, classified as a grade 3c tear. Two hundred thirty (26.3%) women had scarring to the external anal sphincter only, and 176 (20.1%) women had evidence of an external anal sphincter repair.

Tables 1 and 2 show a summary of demographic results.

**Table 1:** Demographics: mean averages and significance

	Mean: Confirmed OASIS (standard deviation)	Mean: No injury (standard deviation)	*P* value
Age (years)	32.1 (4.8)	31.7 (5.0)	= 0.236
Duration of second stage of labour (minutes)	108 (83)	75 (68)	< 0.001*
Baby birthweight (grams)	3509 (491)	3496 (473)	= 0.710
Baby head circumference (cm)	34.5 (1.5)	34.4 (1.4)	= 0.780

Significance denotated by *

**Table 2:** Demographics: percentages and significance

	No injury (percentage)	Confirmed OASIS (percentage)	*P* value
Epidural	55 (23.3%)	369 (42.1%)	< 0.001*
Mediolateral episiotomy	49 (20.8%)	372 (42.5%)	< 0.001*
Instrumental	42 (17.8%)	438 (50.0%)	< 0.001*
Forceps	33 (14.0%)	369 (42.1%)	< 0.001*
Ventouse	8 (3.4%)	65 (7.4%)	< 0.001*
Forceps + Ventouse	1 (0.4%)	4 (0.5%)	= 0.606
Primiparous	197 (83.5%)	697 (80.3%)	= 0.407
Occipitoposterior position	24 (10.2%)	140 (17.7%)	= 0.049*

Significance denotated by *

### Anal endosonography and symptoms

There were 1101 women for whom EAUS and questionnaire data were both available; 870 had a confirmed anal sphincter and 231 did not. Of those with a confirmed anal sphincter injury, 318 (36.6%) had one or more of the following symptoms: flatus incontinence, passive faecal incontinence, urge faecal incontinence and post defecation soiling. Women reported flatus incontinence (277, 31.8%), passive faecal incontinence (15, 1.7%), urge faecal incontinence (51, 5.9%) and post defecation soiling (60, 6.9%). In those with no anal sphincter injury, 65 (28.1%) had one or more of the previously described symptoms; 45 (19.5%) women reported flatus incontinence, 5 (2.2%) passive faecal incontinence, 17 (7.4%) urge faecal incontinence and 14 (6.1%) post defecation soiling.

The incidence of flatus incontinence was significantly greater (*p* < 0.001) in those with a confirmed OASIS compared to those with no confirmed injury. However, there was no significant difference between those with or without anal sphincter injury amongst women who reported passive faecal incontinence, urge faecal incontinence or post defecation soiling.

The mean SMIS in those with a confirmed OASIS was 1.92 (95% CI 1.72–2.14), which was significantly higher (*p* = 0.002) compared to those where injury was not confirmed who had a mean SMIS of 1.36 (95% CI 1.06–1.66).

See Table 3 for a summary of findings.

**Table 3:** Rectal symptoms and significance

	No injury (percentage)	Confirmed OASIS (percentage)	*P* value
Any symptoms	65 (28.1%)	318 (36.3%)	= 0.017*
Flatus incontinence	45 (19.5%)	277 (31.6%)	< 0.001*
Passive faecal incontinence	5 (2.2.%)	15 (1.7%)	= 0.656
Urge faecal incontinence	17 (7.4%)	51 (5.8%)	= 0.401
Post defecation soiling	14 (6.1%)	60 (6.8%)	= 0.652
St Mark’s Incontinence score	1 (1.36 to 2 d.p.)	2 (1.92 to 2 d.p.)	= 0.002*

Significance denotated by *

### Anal endosonography and anorectal physiology

One thousand eighteen women returned for ARP tests. The mean maximal resting pressure was 52.9 (± 20.2) mmHg, and the mean incremental squeeze pressure was 49.1 (± 29.5) mmHg. The mean total pressure was 102 (± 37.9) mmHg.

The expected normal range for mean maximal resting pressure is between 50 and 120 mmHg. Both the overall mean and most women, 54.2% (552 out of 1018), fell within that range. However, a large percentage, 45.6% (464 women), had mean maximal resting pressure < 50 mmHg.

Two (0.2%) women had resting pressure > 120 mmHg. The expected normal value for mean incremental squeeze pressure is ≥ 60 mmHg. Three hundred eighteen (31.2%) women had values within the normal range, but the majority, 700 (68.8%) women, had squeezes below the normal value. The normal mean total pressure value is ≥ 110 mmHg; 398 (39.1%) women had total pressures above this value, but the majority 620 (60.1%) had reduced mean total pressures.

Of those that underwent ARP, 838 (82.3%) had evidence of sphincter injury on EAUS, 179 (17.6%) had no evidence of sphincter injury, and 1 (0.1%) did not undergo EAUS testing. In those with an OASIS, the mean maximal resting pressure was 51.5 mmHg, with a mean incremental squeeze pressure of 46.4 mmHg. These were statistically lower (*p* < 0.001) than both the mean maximal resting pressure and the mean incremental squeeze pressure of those with no anal sphincter injuries, who had pressure values of 59.4 mmHg and 62.0 mmHg, respectively.

In those with a defect to the external sphincter only, the mean maximal resting pressure was 50.0 mmHg, which was not significantly different (*p* = 0.528) than those with an internal sphincter defect who had a mean maximal resting pressure of 47.2 mmHg. However, the mean incremental squeeze pressure in those with an isolated defect to the external sphincter was 45.8 mmHg, which was significantly higher (*p* = 0.037) than those with an internal sphincter defect, who had a mean incremental squeeze pressure of 41.0 mmHg.

Neither the mean maximal resting pressure nor the mean incremental squeeze pressure was significantly different between those with persistent external sphincter defects when compared with those who had evidence of a repair (*p* = 0.470 and *p* = 0.832, respectively). The mean maximal resting pressure in those with evidence of a repair was 51.4 mmHg and the mean incremental squeeze pressure was 46.4 mmHg. This was, however, found to be significantly higher than the average pressures in those with persistent internal sphincter defects (*p* = 0.034 and *p* = 0.042, respectively) and significantly lower than those who had scarring alone (*p* = 0.005 and p = 0.042, respectively).

The mean maximal resting pressure in those with scarring only was 57.4 mmHg, and the mean incremental squeeze pressure was 52.6 mmHg. These pressures were significantly higher than both isolated external sphincter defects (*p* < 0.001 and *p* = 0.012, respectively) and those with persistent internal sphincter defects (*p* < 0.001 for both pressures).

See Tables 4 and 5 for a summary of findings.

**Table 4:** Mean sphincter pressures by injury type

Injury type (number)	Mean maximal resting pressure in mmHg (range)	Mean incremental squeeze pressure in mmHg (range)	Mean total pressure in mmHg (range)
Everyone (1017)	52.9 (9–130)	49.1 (0–187)	102.0 (25–254)
No anal sphincter injury confirmed (179)	59.4 (16–121)	62.0 (0–187)	121.4 (39–254)
Confirmed anal sphincter injury (838)	51.5 (9–130)	46.4 (0–165)	97.9 (25–238)
Isolated external anal sphincter defect (220)	50.0 (10–103)	45.8 (4–129)	95.8 (25–199)
Internal anal sphincter defect (233)	47.2 (9–110)	41.0 (1–117)	88.3 (28–196)
Repair (167)	51.4 (18–130)	46.4 (0–158)	97.8 (29–226)
Scarring (216)	57.4 (13–116)	52.6 (4–165)	110.0 (29–238)

**Table 5:** Pressure significance between injury types

	Significance (*p* value)		
	Mean maximal resting pressure	Mean incremental squeeze pressure	Total pressure
Injury vs. no injury	< 0.001*	< 0.001*	< 0.001*
External defect vs. internal defect	0.528	0.037*	0.017*
External defect vs. repair	0.470	0.832	0.562
External defect vs. scarring	< 0.001*	0.012*	< 0.001*
Repair vs. internal defect	0.034*	0.042*	0.001*
Repair vs. scarring	0.005*	0.042*	0.006*
Scarring vs. internal defect	< 0.001*	< 0.001*	< 0.001*

Significance denotated by *

## Discussion

The incidence rates of OASI have increased over recent years, from 1.8% to 5.9% between 2002 and 2012 in England, and this is thought to be due in part to better recognition and reporting [2]. Correct diagnosis and timely repair of anal sphincter injuries are extremely important in limiting the occurrence of bowel symptoms, some of which may not develop for many years after the injury occurred. One recent study which compared women who were diagnosed and received a primary repair at the time of delivery, and women whose OASIS were missed at delivery found that women with missed tears had significantly worse anal incontinence and urinary function scores [21].

Women who have sustained a previous OASIS and have further pregnancies should be given careful guidance regarding future mode of delivery. There are no systematic reviews from which to form a more definitive protocol regarding future deliveries; however, a recent multi-centre cohort study found women with previous OASIS to be at increased risk of future injuries with a reoccurrence rate to be 10.2% [22]. A study observing vaginal delivery after OASIS found reduced squeeze pressures 3 months post-partum compared to caesarean section; however, this did not translate to increase anorectal symptoms [23]. More research is needed in this area to determine which women are at a high risk and can, therefore, be advised appropriately. We did not have the information available in our study to calculate a recurrence rate; however, this is an area of potential further follow-up study.

Our study reports the outcome following anal endosonography in women who have a clinically diagnosed OASIS and describes the contributing factors that increase the risk of having bowel symptoms and reduced anal squeeze pressures.

The prevalence of anal incontinence following primary repair of OASIS is thought to be between 15% and 61%. This can have serious social, psychological and physical consequences, which can lead to women having to alter their lifestyle or wear a pad or plug, highlighting the need for accurate primary repair [24]. We found that the most reported symptom amongst our cohort was flatus incontinence, with nearly a third of women reporting this. Women who reported flatus incontinence were significantly more likely to have an OASIS confirmed on EAUS than those who did not. This finding was not however replicated for urge faecal incontinence, passive faecal incontinence or post defecation soiling. There was also a significant increase in SIMS amongst those with an EAUS-confirmed OASIS, a finding that has been replicated in other studies [25, 26].

Several statistically significant findings were made in relation to the analysis of EAUS and ARP. Multiple different analyses were carried out comparing the different injuries and their effects on mean maximal resting pressure, mean incremental squeeze pressure and the total pressure. All three pressures were significantly reduced in those with a confirmed injury when compared to those without injury, which is like results reported elsewhere [25]. When comparing those with isolated external sphincter injuries and those with internal sphincter injuries, the mean incremental squeeze pressure and mean total pressures were found to be significantly lower amongst the group with internal sphincter injuries. These results were also found in another study which looked at similar outcomes; however, they also found that the mean maximal resting pressure was significantly reduced in the group with internal sphincter injuries while our results did not replicate this [27]. Whilst this study was useful to compare to our own, it is important to note that the sample size in this study was quite small, with only ten participants with isolated external sphincter injuries and six with internal sphincter injuries.

There was no significant difference between the squeeze pressures of those with persistent external sphincter defects and those with evidence of repairs, suggesting that primary repair was not effective in restoring function. However, as mentioned above, a study looking into missed OASIS found these patients suffered more rectal and urinary symptoms than those who had undergone primary repair at the time of delivery [21].

When reviewing studies looking into the long-term effects of OASIS, it is a common finding that the higher the grade of tear the worse the outcome. One such study, which had a mean follow-up time of 6.6 years, found that those with tears classified as 3c/4 had significantly more anal incontinence symptoms and lower anorectal physiology pressures than those with 3a/3b tears [28]. Another long-term study, which had a minimum follow-up of 10 years, found a significant reduction in quality of life amongst those who suffered an OASIS compared to those who had an uncomplicated vaginal delivery [29]. It is, however, important to be aware of the potential impact of selection bias in long-term follow-up studies as those with more severe symptoms are more likely to respond than some who have suffered limited or no ill effects [30].

One limitation of this study is its retrospective nature. This also meant that a comparative sample of patients without clinically diagnosed OASIS could not be identified over a similar time period for comparison of demographic data. Besides this, despite the large sample size, this was a single-centre study.

## Conclusion

EAUS remains the gold standard assessment for obstetric anal sphincter injuries and 21.2% of the women with a suspected OASIS in this study can be reassured that they do not have an injury. This information is important for women in planning future mode of delivery and allows them to avoid potentially unnecessary caesarean sections. EAUS, along with ARP, has also allowed us to show that the anal physiology results in women with no injury are higher than those with an OASIS.

Bowel symptoms were reported by 34.8% of women with a clinically diagnosed obstetric anal sphincter injury. Those with confirmed injury on EAUS are more likely to complain of flatus incontinence and anal sphincter pressures were significantly reduced.

Long-term follow-up studies are required in the assessment of the long-term impact of injuries and future mode of delivery.
